# Rosette forming glioneuronal tumor in association with Noonan syndrome: pathobiological implications 

**DOI:** 10.5414/NP300374

**Published:** 2011-10-18

**Authors:** M. Karafin, G.I. Jallo, M. Ayars, C.G. Eberhart, F.J. Rodriguez

**Affiliations:** 1 Department of Pathology, Division of Neuropathology; 2 Neurosurgery, Johns Hopkins University, Baltimore, MD, USA

**Keywords:** Noonan syndrome, glioneuronal tumor, rosette forming glioneuronal tumor, RAS, MAPK/ERK

## Abstract

Noonan syndrome, a distinctive syndrome characterized by dysmorphism, cardiac abnormalities and developmental delay, has been associated with a number of malignancies, however, only a few cases of primary glial or glioneuronal neoplasms have been reported. We report here the case of an 18-year-old with Noonan syndrome who developed a rosette forming glioneuronal tumor of the posterior fossa. The tumor demonstrated strong pERK immunoreactivity, suggesting MAPK/ERK pathway activation. Molecular testing did not reveal *BRAF* rearrangements (fusion transcripts) by PCR or a *BRAF* ^V600E^ mutation by sequencing. We review the literature regarding the molecular pathogenesis of Noonan syndrome and primary brain tumors, and consider the intriguing link between their common molecular pathways.

## Introduction 

Noonan syndrome is a heterogeneous autosomal disorder that was first described by Jacqueline Noonan [1]. This disorder is characterized by a combination of unique facial features, short stature, cardiac abnormalities, chest wall abnormalities and developmental delay [2]. There are five genes currently associated with Noonan syndrome. These genes are all known to be part of the Ras pathway, which is also implicated in a number of neoplastic processes. We describe below a young patient with Noonan syndrome and a rosette forming glioneuronal tumor of the 4^th^ ventricle, an association not previously reported to our knowledge. We discuss the current literature regarding the pathogenesis of Noonan syndrome and suggest a common molecular underpinning for both primary astrocytic or glioneuronal tumors and Noonan syndrome. 

## Case history 

**Figure 1.**
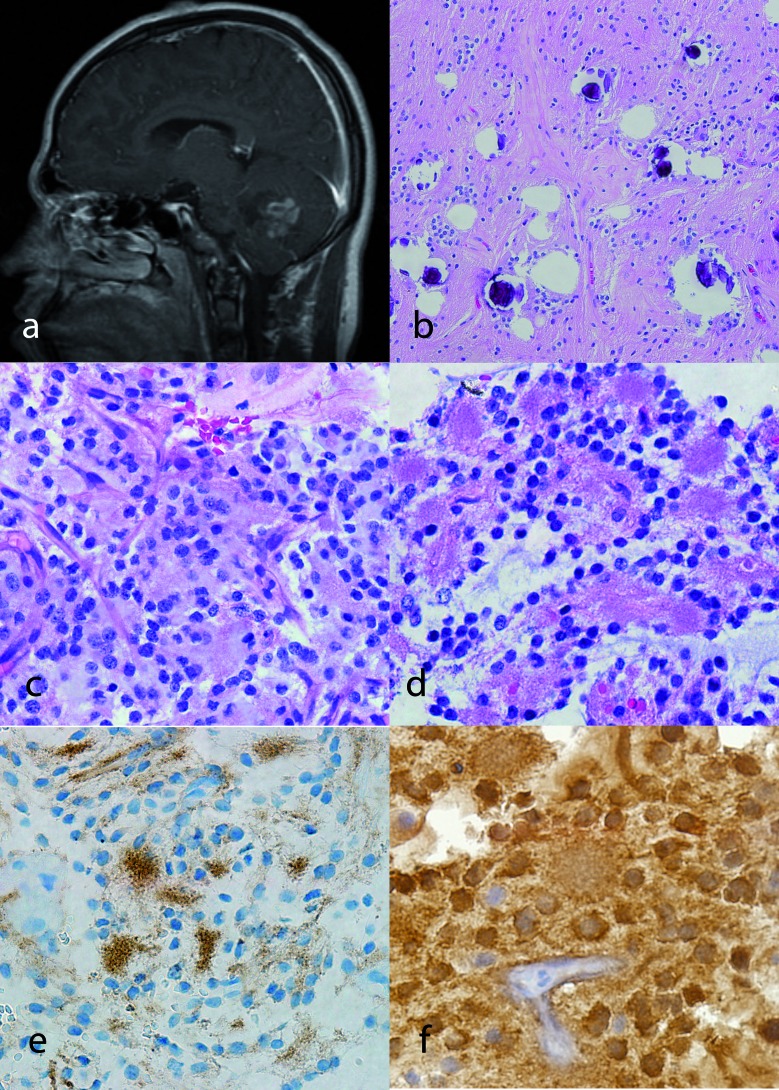
A well circumscribed enhancing neoplasm involving the vermis and 4^th^ ventricle was present (Sagittal T1-weighted MR (a). Microcalcifications were also present on histologic examination (b). The neoplasm was lobulated and low grade with round to oval cells reminiscent of pilocytic astrocytoma (c). In addition distinctive rosettes reflecting neuronal differentiation were present (d). Synaptophysin was strongly expressed in rosettes and perivascular pseudorosettes (e). Immunoreactivity for pERK was strong and diffuse (f).

An 18-year-old male with the clinical features characteristic of Noonan syndrome including developmental delay and mild pulmonary valve stenosis, presented to the Johns Hopkins Hospital in August 2008 with a scalp lesion. His Noonan syndrome diagnosis was based on established clinical criteria [22], as well as a positive family history including his father. Mutational testing was not performed. He had no previously documented cancers. Prior to this admission, his primary care physician ordered an MRI of his brain to evaluate the scalp lesion, and a posterior fossa tumor was incidentally identified. MRI revealed that the mass arose in the vermis, and occupied the fourth ventricle (Figure 1a). The scalp lesion demonstrated minimal bone scalloping. Surgical resection of both lesions was recommended, and a gross total resection of the posterior fossa lesion was accomplished. He has since had no recurrence of his brain tumor, after a follow-up of 21 months. 

## Pathology 

The neoplasm demonstrated variable cytology, including cells with elongated nuclei and others with round, oligodendroglia-like quality (Figure 1b). Glomeruloid vasculature was present, and mitotic activity was low. The combination of findings was reminiscent of a low grade glioma. A distinctive focal finding was the presence of small rosettes with delicate cores (Figure 1d). 

Immunohistochemical stains demonstrated GFAP expression in much of the tumor, while synaptophysin labeled the small rosettes (Figure 1e) as well as perivascular processes. The combination of findings was most compatible with a rosette forming glioneuronal tumor of the 4^th^ ventricle. The scalp lesion was diagnosed as a vascular malformation, and consisted of thin-walled vessels composed of CD34 positive, D240 negative endothelial cells. 

MAPK/ERK activation in specific tumor types may be inferred using antibodies detecting phosphorylated components of the pathway [3]. Immunohistochemical staining for pERK was performed using a rabbit monoclone on antibody ((D13.14.4E)XP^®^ Cell Signaling Technology) at a dilution of 1 : 100, which demonstrated strong cytoplasmic and nuclear immunoreactivity suggesting MAPK/ERK pathway activation in the majority of tumor cells, including the rosettes (Figure 1f). Molecular testing by PCR using primers designed to detect the three known *BRAF:KIAA1549* fusion transcripts was negative. Sequencing of *BRAF* exons 15 and 11 was also negative for *BRAF* ^V600E^ or *BRAF* ^G648A^ mutations, respectively. 

## Discussion 

Noonan syndrome is a congenital syndrome with an estimated incidence of 1 : 1,000 to 1 : 2,500 live births [2]. It can be inherited in an autosomal-dominant manner, or, as is true in about 60% of cases, can be acquired sporadically [4]. According to the Van Der Burgt criteria developed in1994, the diagnosis of Noonan syndrome requires typical facial features with either one other major criteria, including pulmonary valve stenosis and short height, or two minor criteria, such as developmental delay. The patient had pulmonary valve stenosis (major criteria) and typical facial features (major criteria) with developmental delay (minor criteria), fulfilling the diagnostic criteria for Noonan syndrome [5]. 

The genetics of Noonan syndrome has not been completely elucidated, but is known to derive its pathogenesis from at least five genes [6]. Over 50% of cases of Noonan syndrome involve a mutation in the *PTPN11* gene [2]. This gene codes for a nonreceptor protein tyrosine phosphatase SHP2 [7]. Mutations at this gene in Noonan syndrome lead to a gain-of-function mutation of the SHP2 protein leading to an increase in signal transduction through the Ras-MAP kinase pathway [2]. Interestingly, the remaining genes found to result in the clinical features of Noonan syndrome also involve the Ras-MAP kinase signaling system, including mutations in *KRAS *[8], *SOS1 *[9] and *RAF1 *[6, 10]. Mutations of the *NF1* gene have also been shown to cause Noonan syndrome, in addition to the more frequent syndrome, neurofibromatosis Type 1. Perhaps not surprisingly, patients with Noonan syndrome attributed to a mutation at the *NF1* gene also have findings consistent with neurofibromatosis [11]. 

The patient was diagnosed with a primary low grade brain neoplasm at 18 years of age. The relationship between Noonan syndrome and neoplasia has been documented in the literature. Noonan syndrome has been most associated with hematological malignancies such as juvenile myelomonocytic leukemia, myelodysplastic syndrome, B-cell acute lymphoblastic leukemia, acute myelogenous leukemia, but the incidence is low [12]. Bentires-Alj et al. [12] reported cases of lung cancer, melanoma, and colon cancer in patients with *PTPN11* mutations. Primary brain tumors, in particularly astrocytomas, have been described in a handful of cases of Noonan syndrome (Table 1). It is of interest that most of these tumors represent pilocytic astrocytomas, a neoplasm characterized at the genetic level by aberrations leading to increase MAPK/ERK pathway signaling [13, 14]. Pilocytic astrocytoma is also the most frequent brain tumor arising in the setting of NF1 [15], another genetic syndrome resulting in constitutive MAPK/ERK pathway activation. 

The current report is unique in that a patient satisfying clinical criteria for Noonan syndrome developed a rosette forming glioneuronal tumor of the fourth ventricle. This neoplasm is a new addition to the WHO classification of brain tumors [16]. Histologically, it is a biphasic tumor composed of a well differentiated astrocytic component, and a neuronal component characterized by rosettes and perivascular pseudorosettes [17]. Although the genetic features of this tumor are not well established given its rarity, the presence of an astrocytic component resembling pilocytic astrocytoma, which often predominates, suggests that MAPK/ERK activation may play a role. In addition, a rosette forming glioneuronal tumor has been previously reported in a patient with NF1 [18], as well as one prior report of a Noonan syndrome patient with a disseminated glioneuronal tumor in leptomeninges [19]. 

The realization that the Ras pathway is upregulated both in Noonan syndrome and in sporadic cases of low grade glial/glioneuronal tumors increases the likelihood that the neoplasm identified both in our case report and in other previous studies did not occur by chance. However, much remains to be learned about how the upregulation of this pathway relates to Noonan syndrome and carcinogenesis in general. Additional studies both from those who study brain tumors and congenital diseases such as Noonan syndrome are clearly needed, given the fact that inhibitors of this pathway have been developed [20]. 

**Table 1.** Cases of Noonan syndrome patients with low grade glial and glioneuronal tumors.

Case	Age at diagnosis	Gender	Location	Pathology	Mutation
Sanford et al. 1999 [21]	16	M	Cervicomedullary junction	Pilocytic astrocytoma	Unknown
Jongmans et al. 2004 [22]	18	F	Hypothalamus	“Low grade glioma”	*PTPN11*
Sherman et al. 2009 [19]	6	M	Suprasellar/Diffusely in leptomeninges	Low grade glioneuronal tumor	*PTPN11*
Fryssira et al. 2008 [1]	11	F	Sellar/suprasellar region	Pilocytic astrocytoma	Unknown
Schuettpelz et al. 2009 [23]	8	M	Suprasellar	Pilocytic astrocytoma	*PTPN11*
Current case	18	M	4^th^ ventricle	Rosette forming glioneuronal tumor of the 4^th^ ventricle	Unknown
